# Supplementary material for: Nanoscale thermal control of a single living cell enabled by diamond heater-thermometer
Source: Sci Rep. 2023 May 26;13:8546. doi: 10.1038/s41598-023-35141-4 (PMC10220034; doi:10.1038/s41598-023-35141-4)
Supplement: Supplementary file 1 — Supplementary Figures. [file 41598_2023_35141_MOESM1_ESM.pdf]

Nanoscale thermal control of a single living cell enabled by diamond heater-thermometer

Supplementary Information

Generally, the measured temperature is a product of non-linear least squares regression and its minimum detectable variation is naturally limited by errors in emission peak fitting which arise due to 1) finite spectral resolution and 2) insufficient signal to noise ratio. While the former contribution decreases as $(n - p)^{-1/2}$, where n is the dataset size and p is the number of independent parameters to be determined and is negligible in our case ($n = 15, p = 3$)[1], the latter is defined by acquisition time and plays a crucial role. So, one can see from **Fig. S3** the standard deviation $\sigma_{\lambda_{center}}$ ~ 0.0013 nm (0.12 °C) obtained at 10 s acquisition is ~3 times less than that for acquisition 1s shown in Fig. 3c.

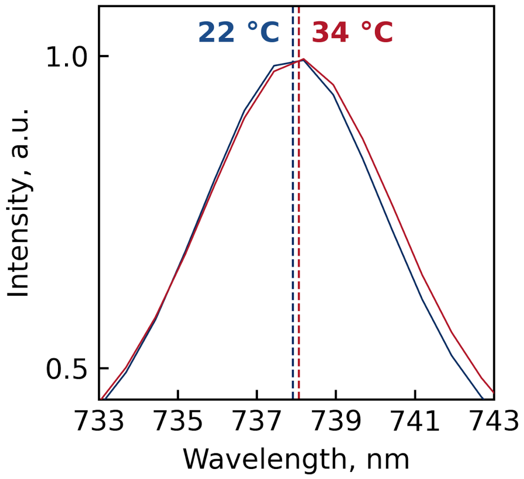

Figure S1. The fluorescence spectra recorded at 0.2 mW and 4.8 mW power corresponding to 22 °C (blue) and 34 °C (red) heating temperatures with spectral positions 737.906 nm and 738.029 nm respectively.

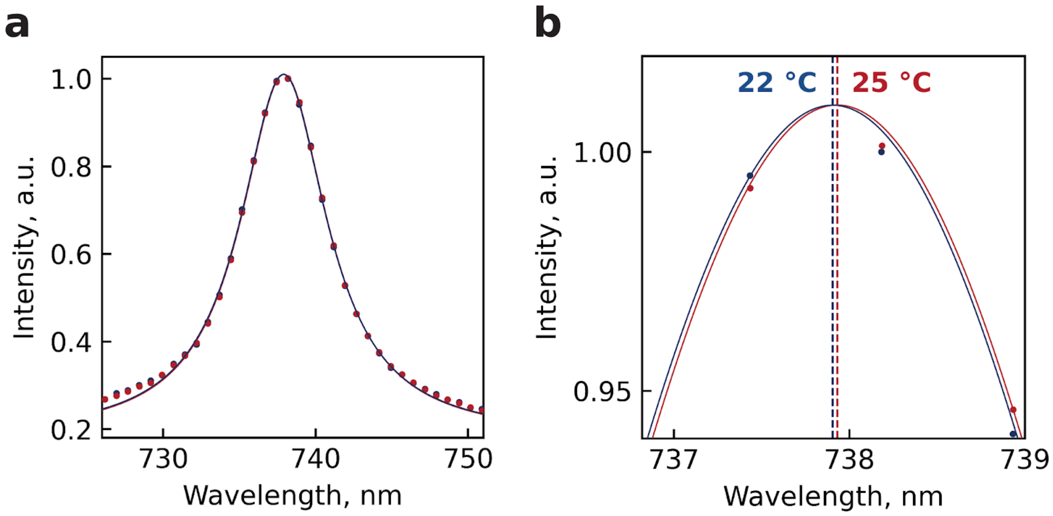

Figure S2. (a)-(b) The fluorescence spectra recorded at 0.2 mW and 1.2 mW power corresponding to 22 °C (blue) and 24.6 °C (red) heating temperatures with spectral positions 737.906 nm and 737.932 nm respectively. The scatters represent spectrometer data, the solids are the approximation with the Lorentzian profile. Note, (b) shows the same spectra in enlarged scale.

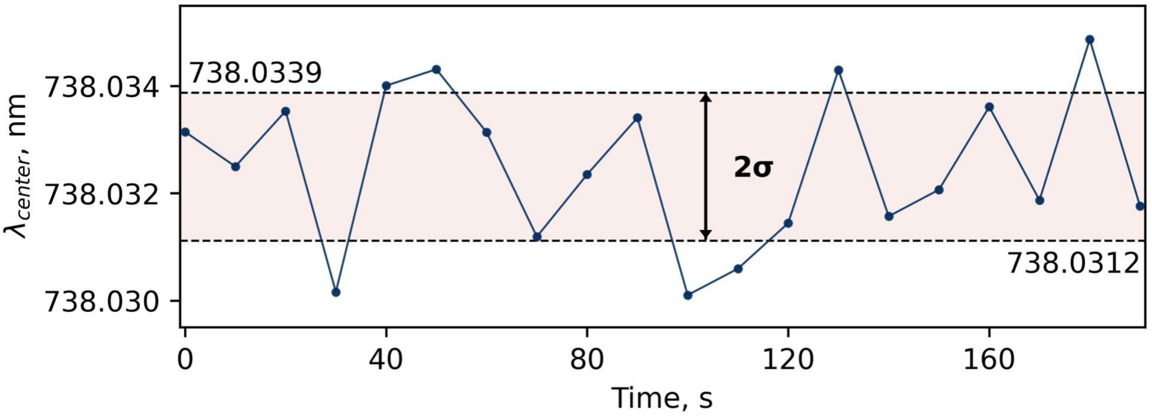

Figure S3. Time-evolution of spectral positions extracted using Levenberg-Marquardt method from twenty spectra which were recorded with 10s acquisition at 4.8 mW power. The light red area extends the double standard deviation 2σ, where $\sigma = 0.0013$ nm corresponding to 0.12 °C in temperature equivalent.

1. Wolberg, J. Data analysis using the method of least squares: extracting the most information from experiments. *Springer Science & Business Media*, (2006).
